# Encyclopædia Medica

**Published:** 1902-06

**Authors:** 


### Reviews of Books.

**Encyclopædia Medica.** Under the general editorship of Chalmers Watson, M.B. Vols. IV.—IX.: *Foot—Pregnancy*. Edinburgh: William Green & Sons. 1900–1901.

These volumes, each containing between five and six hundred pages, are a continuation of those formerly reviewed (*vide* vols. xvii. and xviii.). Some thirty to forty principal articles are to be found in each volume, and these, written by authorities on the respective subjects, are important monographs. In a comprehensive work like this there must of necessity be some inequality, but for the most part the editor has successfully avoided this difficulty. Other encyclopædias devoted to medicine, surgery, and gynæcology respectively contain fuller details; but, for their size, the present volumes contain more information than other works of a similar class.

It is difficult to select individual articles for comment, but that on gout, written by the editor (vol. iv.), cannot fail to be full of interest and instruction to the reader. It extends over nearly fifty pages, and gives considerable detail on the dietetic questions associated therewith. He has little belief in drug treatment: that is surprising, for we have always found that an attack of acute gout is more amenable to drugs than many other inflammations, and salicylates are nearly as valuable as in the allied rheumatic arthritis. He gives a caution as to the use of colchicum; but why such reiterated cautions as to the use of this drug should be needful we have always been at a loss to understand. There does not seem to be any mention of a class of drugs which do appear to increase the elimination of uric acid and urates; such drugs as piperazine and urosine (quinate of lithia) seem likely to be of much service.

Mr. D'Arcy Power treats of fractures; Mr. Mayo Robson, in conjunction with Dr. Macrae, deals with the gall-bladder and bile ducts; and to Mr. Moynihan is assigned the subject of hernia. All these are dealt with in the thorough manner which we should expect from their distinguished authors. We should, however, like to enter a protest against the statement that "there can be no question" that Bassini's operation for hernia is "incomparably the most successful in results," for there are many moderate cases of rupture which are far better treated by the less severe operation of Kocher.

A very noteworthy article is that on invalid feeding (vol. v.), written by Mrs. Chalmers Watson, M.D., who remarks that every practitioner should possess some knowledge of cookery. It is pointed out that the effect of different degrees of heat on albumin is the keynote to all the various culinary methods, which are variations of one another depending on whether the meat juices are to be retained or extracted. The cooking of vegetables is commonly considered to be mere detail; but as the nutritive value and digestibility of vegetable foods depend largely on their careful preparation, a short account of the best methods of preparing many of them is here given. The article contains a large number of formulæ for the cooking of delicacies for the invalid and the aged: the diabetic receives special attention, and a series of *menus* for six days gives a very large variety of attractive dishes.

The subject of general insanity receives (vol. v.) consideration under four headings — etiology, pathology, nature and symptoms, and treatment,— these sections being furnished respectively by Drs. Urquhart, Ford Robertson, G. R. Wilson, and Mercier. It is difficult to conceive work better done, within the allotted space, than that by these authors, each of whom has successfully achieved condensation without detriment to easy reading or suppression of important matter. In Dr. Robertson's article the practitioner will find an astonishingly comprehensive and up-to-date summary of the special pathology of mental disease, which he may well trouble himself to thoroughly digest. The section on the nature and symptoms of insanity is excellent, and equally displays powers of observation, originality and clearness in expression. Although we have not yet met with writing on this subject more readily intelligible to the general medical reader, there is much in Dr. Wilson's contribution that will repay careful perusal by those making a special study of insanity. The section on general and special treatment contains a short and clear *resumé* of the means for effecting the control of an insane person under various circumstances and the principles which should guide the practitioner in advising as to the necessity of enforced segregation, the methods of control and their proper application, the conditions calling for special treatment, and the details of this both therapeutic and otherwise. All these matters are dealt with in a most able and convincing manner by the author; this by no means lengthy chapter indeed contains an immense amount of valuable information.

An article on life insurance (vol. vi.), by Dr. William Elder, gives a very complete but condensed account of the duties of the medical examiner and the problems which he is called upon to endeavour to solve. These problems are often incapable of solution, and it is the duty of the examiner to give the applicant the benefit of any doubt that may be, unless he has good reason to consider the risk more than should be accepted by any office for which he may be employed. The risks of life are obviously incapable of absolute prediction, and the examiner can only report as to the impression made upon himself by the applicant; but a review of such an article as this leads to the conclusion that medical men should not think lightly of their duties as medical referees to insurance offices, whose commercial prosperity is very closely related to the accuracy and good faith of their medical examiners.

The subject of labour is most comprehensively dealt with in a series of able articles by several authors.

The articles on diseases of the larynx and nose (vols. vi. and viii.) are the work of various authors, the chief contributions being those on "Inflammatory Diseases," by St. Clair Thomson and Tilley; "Chronic Infective Diseases," by McBride; "Malignant Neoplasms," by Sir Felix Semon; "Post-Nasal Growths," by Macdonald; "Neuroses of the Larynx and Nose," by Watson Williams; and "Diseases of the Accessory Nasal Sinuses," by Logan Turner; while examination of the larynx and nose are dealt with by M. Hunt and Cresswell Baber respectively. If we must single out any one contribution for special note it would be that by Sir Felix Semon, for his article is a masterly exposition of the diagnosis and the methods of radical extirpation of the disease in suitable cases, which have been placed in such a satisfactory position mainly by the investigations and good work of this distinguished author. Dr. John Thomson's contributions on congenital laryngeal stridor and laryngismus stridulus are valuable and sound, drawing clear distinctions between these two affections.

Several articles on diseases of the lungs (vol. vii.) are written by Dr. R. W. Philip, of Edinburgh. In phthisis he rightly claims that the therapeutic measures necessary to what he calls "a physiological life" are most conveniently carried out in a sanatorium, and he adds that, having regard to the appalling frequency of the disease, there is room for much larger provision of such sanatoria, and more especially for the poorer classes, including persons in receipt of limited incomes which cease in case of illness. He states that mere change to a better climate is commonly insufficient, and he is in accord with other authorities in his belief that the special curative influence frequently attributed to certain climates cannot be maintained. We are gratified to see that the writer does not ruthlessly sweep away all drugs in the management of tuberculous lungs.

The article on epidemic cerebro-spinal meningitis is by Dr. William Osler, of Baltimore, whose *resumê* of the disease contains a good account of two important aids in diagnosis which have been brought forward of late years—Kernig's sign and lumbar puncture, both of which are of the greatest possible assistance both in the establishment of the malady and its differentiation from allied conditions. One of these is pneumococcic meningitis, inasmuch as the pneumococcus can usually be readily differentiated from the diplococcus intracellularis. The author is inclined to the belief that the lumbar puncture may have some little therapeutic action apart from its diagnostic value.

Mr. H. J. Stiles deals with diseases of the mammary gland in a clear and comprehensive manner. The researches of this observer on the lymphatic supply of the breast have been helpful in placing the modern operation for carcinoma of the mamma on a scientific basis, though Mitchell Banks long before advocated similar methods on clinical grounds. We should like therefore to have seen that Mr. Banks received due credit for his work in this department of surgery amongst the other honourable names mentioned in the text.

Mrs. Garrett Anderson, M.D., writes on neurasthenia (vol. viii.) a very interesting account of a very difficult subject, and as regards diagnosis she shrewdly gives the following advice (p. 333):—"Having found no objective evidence of organic disease, listen to the patient's story, asking no suggestive questions. If a neurasthenic patient is encouraged to narrate his experiences and symptoms, there will, as a rule, be ample evidence against organic disease. No system disease one knows of ever had half the variety of symptoms and the variation of conditions described by the neurasthenic."

One of the most important series of articles is that on paralysis, written by Drs. Risien Russell, Frederick Batten, and James S. Collier (vol. ix.). It extends to one hundred pages, on the system-lesions of the cord and other diseases of the grey and white matter. A very complete chapter on parasites by Dr. W. J. Ritchie, of Edinburgh, follows.

The articles on diseases of the ovaries, by Alban Doran, and diseases of the pancreas, by Mayo Robson, contain much valuable information. The pancreas has only lately come under the hands of the surgeon, and those who are unfamiliar with the brilliant results which have been achieved in cases of chronic pancreatitis would be greatly enlightened by reading Mr. Robson's remarks on this subject.

Enough has been said of the work as a whole to show that it amply fulfils all that we expected of it when dealing with the earlier volumes. The three remaining volumes will complete the series of a reliable, succinct and commendable encyclopædia.

